# Klinefelter syndrome with long-arm X-chromosome deletion

**DOI:** 10.1515/almed-2022-0106

**Published:** 2022-12-19

**Authors:** Vanesa Escribano Hernández, Maria Ángeles Sanz de Benito, Raffaelle Carraro Casieri

**Affiliations:** Department of Clinical Analysis, Hospital Universitario de La Princesa, Madrid, Spain; Service of Endocrinology and Nutrition, Hospital Universitario de La Princesa, Madrid, Spain

**Keywords:** deletion, hypogonadism, Klinefelter syndrome

To the Editor,

Klinefelter syndrome (KS) was first described in 1942 by Harry F. Klinefelter in nine men with testicular abnormalities, failure to produce spermatozoa and gynaecomastia [1], being the first sexual chromosome abnormality to be described. In 1959, Jacobs and Strong found that this phenotype was due to the presence of an extra X chromosome [2].

Klinefelter syndrome (KS) is an ananeuploidy that affects sexual chromosomes characterized by the presence of two X chromosomes and a Y chromosome in subjects with a male phenotype. It is the most common sexual chromosome abnormality, with an incidence of 1 in 600 male births, being the most frequent cause of genetic infertility in males, with a frequency of 4% [3]. Clinically, patients are tall, with narrow shoulders, wide hips, weak muscles, scarce body hair, small testes, gynaecomastia and infertility. Complementary tests revealed aspermatogenesis, reduced levels of serum testosterone, elevated levels of follicle-stimulating hormone (FSH) and luteinizing hormone (LH) and androgen deficiency (hypergonadotropic hypogonadism). It is associated with other comorbidities, including osteoporosis, varicose veins, thromboembolic disease, diabetes, chronic bronchitis, bronchiectasis, emphysema and neoplastic diseases. Phenotypic traits are subtle and generally remain unnoticed during childhood and adolescence. Diagnosis is usually established during puberty due to poor development of secondary sexual characters, or even in adulthood, upon investigation of infertility.

We report the case of a 26-year-old man referred to the Unit of Urology due to the presence of phimosis. Reported pubarche occurred at 12 years, with deepening of voice and growth of facial hair at 18, although it is sparse and needs shaving only once weekly. He experiences erections and sexual desire, and does not show other symptoms.

On physical examination, the patient has a height of 177 cm, a weight of 76.6 kg and an arm span of 180 cm, with mild phimosis, hypotrophic testes and mild gynaecomastia.

Seminogram reveals a volume of sperm of 3.2 mL, translucent, with total liquefaction at 1 h and normal viscosity, with a pH of 8.5 azoospermia.

Scrotal ultrasound demonstrates a reduction of the size of the testes, diffuse hypogenicity and a hyperechogenic point related to microcalcifications, without intraparenchymal lesions. Epididymes with preserved morphology, with an 8-mm cyst in the head of the left epididymis. Hydrocele or varicocele not noted.

Findings related to the hormonal profile of the patient are shown in Table 1.

**Table 1:** Hormonal profiles of patients at diagnosis.

	20/05/2016	19/10/2016	Reference range	Units
LH	13.46	13.75	0.57–12.07	mUI/mL
FSH	33.23	28.63	0.95–11.95	mUI/mL
PRL	25.93	24.35	3.46.19.40	ng/mL
Estradiol		14	11–44	pg/mL
Progesterone		0.3	0.1–0.2	ng/mL
Testosterone	1.99	1.83	2.40–8.70	ng/mL
Calculated free testosterone		146.8	228–720	pmol/L
Androgen index		27.95		
SHBG		22.7	14.5–48.4	nmol/L

LH, luteinizing hormone; FSH, follicle-stimulating hormone; SHBG, sex hormone binding globulin. Free testosterone estimation method: Vermeulen equation. Androgen index estimation method: [Total testosterone (nmol/L)/SHBG (nmol/L)] * 100.

Upon diagnosis of primary hypergonadotropic hypogonadism (reduced testosterone with elevated LH and FSH concentrations), the patient was referred to the Genetic Counseling Unit. Cytogenetic test revealed the presence of 47 chromosomes in all metaphases, with an XXY chromosome and subcentromeric long-arm X-chromosome deletion. Comparative genomic hybridisation (CGH array) was performed with a male control (Promega) on the KaryoArray^®^v3.0 system (Agilent), with a mean resolution of 1 oligo every 9 kb in the regions of greatest interest (detection of >25 kb gains or losses) and of 175 kb in the rest of the regions (detection of >400 kb losses or gains). The result is a genomic pattern corresponding to male sex and an arr [hg19] Xp22.33 q13.3 (60,679–74,057,587)×2 formula. Two copies were found of an approximate interval of 74 Mb located at X-chromosome region p.22.33–q13.3, being the total number of copies in males 1. Genetic findings explain the phenotype of the patient.

Follow-up is performed by the Unit of Endocrinology, where androgen replacement therapy is prescribed.

Almost 80% of patients with KS have a 47,XXY karyotype, Of them, 50% receive the extra X chromosome from their father, whereas the other 50% received it through maternal inheritance. When inherited from the mother, the error occurs during meiosis I o II or in the first mitotic division of embryonic development. Conversely, in aberrations inherited from the father, the error always occurs during meiosis. The remaining 20% of patients have other abnormalities in the number of copies of sexual chromosomes (48,XXXY; 48,XXYY; 49,XXXXY), 46,XY/47XXY mosaicisms or structural abnormalities in the X chromosome [4].

There are few case reports of KS patients with abnormalities in one of the X chromosomes. Frühmesser et al. [5] found five cases in the literature with deletions in one of the X chromosomes. In 2015, Liang et al. [6] reported the case of a 24-year-old male with infertility as initial symptom whose genetic tests revealed a KS-related aneuploidy due to an extra X chromosome of maternal inheritance. The phenotypic characteristics and genotypes of patients reported in the literature and of the case reported in this study are summarized in Table 2.

**Table 2:** Karyotype and phenotypic characteristics of patients with KS and chromosome-X deletion described by Frühmesser et al. and Liang et al. and the case reported in this study.

Author	Karyotype	Age, years	Height, cm	Weight, kg	Gynaecomastia	Testicular hypotrophy	FSH	LH	Testosterone
Nielsen 1966	47,XY,der (X) [63]/46,XY [6]	54	160	–	NO	YES	–	–	–
Chandra et al. 1971	47,XXq-Y	–	Tall	–	SI	YES	–	–	–
Nielsen 1976	47,X,del (X) (p11→q13:q21→q24),del (Y) (q11)	18	163.5	52.9	NO	YES	↑	↑	Normal
Patil et al. 1981	47,XY,del (X) (pter→q22:)	19	181	67.5	YES	YES	↑	↑	↓
Fryns 1981	47,XY,+del (Xp11)	30	172	59	NO	YES	↑	Normal	↓
Lian et al. 2015	47,XY,+der (X) t (X;18) (p11.4;p11.22)	24	179	66	–	YES	↑	↑	↓
Our case	47,XXY,del (X) (q13.3;qter)	26	177	76.6	YES	YES	↑	↑	↓

## References

[1] Klinefelter HF, Reifenstein EC, Albright F (1942). Syndrome characterized by gynaecomastia, aspermatogenesis without leydigism, increased excretion of follicle stimulating hormone. J Clin Endocrinol.

[2] Jacobs PA, Strong JA (1959). A case of human intersexuality having a posible XXY sex-determinating mechanism. Nature.

[3] Groth KA, Skakkebæk A, Høst C, Gravholt CH, Bojesen A (2013). Clinical review: Klinefelter syndrome--a clinical update. J Clin Endocrinol Metab.

[4] Lanfranco F, Kamischke A, Zitzmann M, Nieschlag E (2004). Klinefelter’s syndrome. Lancet.

[5] Frühmesser A, Kotzot D (2011). Chromosomal variants in Klinefelter syndrome. Sex Dev.

[6] Lian J, Zhang Y, Wang R (2015). 47,XY,+der (X) t (X;18) (p11.4;p11.22): a unique aneuploidy associated with klinefelter syndrome due to an extra derivative X chromosome inherited maternally. Cytogenet Genome Res.

